# Postural stability disorders—early signs of aging—in physically non-active prisoners

**DOI:** 10.7717/peerj.12489

**Published:** 2022-01-10

**Authors:** Piotr Łapiński, Aleksandra Truszczyńska-Baszak, Justyna Drzał-Grabiec, Adam Tarnowski

**Affiliations:** 1 Academy of Justice, Warsaw, Mazovian, Poland; 2 Faculty of Rehabilitation, Józef Piłsudski University of Physical Education in Warsaw, Warsaw, Poland; 3 Institute of Physiotherapy, Rzeszow University, Rzeszów, Podkarpackie, Poland; 4 Institute of Psychology, Nicolaus Copernicus University in Toruń, Toruń, Poland

**Keywords:** Postural stability, Balance disorders, Prisoners, Physical fitness

## Abstract

**Background:**

There is a need for a study of possible relationship between serving a prison sentence and developing postural stability dysfunction. The aim of the study was to analyze postural stability of physically inactive prisoners. The study group consisted of 24 male prisoners aged 34.6 ± 7.02 years, imprisoned in closed prison and 30 healthy, non-active physically, aged 36.9 ± 7.5 years, who consisted control group. The subjects were imprisoned for a mean of 105.43 ± 58.48 months.

**Methods:**

The static balance test was conducted on bi-modular stabilometric platform CQStab2P.

**Results:**

We found statistically significant differences in several stability parameters. Prisoners results were significantly worse in parameters measured with eyes open: MA (mean amplitude *p* < 0.01), MAAP (mean amplitude in anterio–posterior plane *p* < 0.03), MAML (mean amplitude in medio—lateral plane *p* < 0.04), MaxAP (maximal sway in AP *p* < 0.01), MaxML (*p* < 0.01). With eyes closed the prisoner’s results were significantly worse in SPML (sway path in medio-lateral plane *p* = 0.01), better in MAML (*p* < 0.01) and MaxML (*p* < 0.01), and faster in MVML (mean velocity in medio-lateral plane *p* < 0.01).

**Conclusions:**

(1) Diagnostics aimed at early diagnoses of ageing symptoms should be performed in prisons. It would allow for better prisoner management in terms of assessment of ability to work, free time activity offer and falls prevention. (2) In prisons, in addition to counteracting the typical causes of balance disorders, action should be taken to counteract the causes for balance disorders typical for prison environment, *inter alia*: sensory deprivation—by implementing programmes comprehensively activating prisoners, and hypokinesis—by implementing physical activity programmes that cater for the needs of older prisoners.

## Introduction

Living conditions in most prisons around the world are unhealthy (Coyle, 2007; Pont & Harding, 2019). The reason for this is not only the fact that people with an overrepresentation of diseases and behaviors harmful to health are sent to prisons (WHO Regional Office for Europe, 2019; Fazel & Baillargeon, 2011). Serious factors threatening the health of prisoners are their social isolation and limitation of their living space, which causes numerous deprivations (Shalev, 2014, De Viggiani, 2007), including an limitation of physical activity (Kosendiak & Trzeciak, 2019). Reduced daily physical activity of prisoners is also a result of what constitutes the essence of imprisonment, *i.e*. a significant limitation of prisoners’ ability to decide about themselves. This applies to many normal everyday activities, including ways to use your free time. The higher the level of isolation and supervision applied to prisoners, the less opportunities they have for engaging in leisure and work activities (Zanetti & Macrí, 2020). On the basis of the Central Database of Deprived of Freedom, it was established that in Poland 97.14% of the prisoner population were people aged 18–64. According to the latest recommendations, these people should undertake at least 150–300 min of moderate-intensity aerobic physical activity (or at least 75–150 min of vigorous-intensity aerobic physical activity) and also muscle-strengthening activities at moderate or greater intensity that involve all major muscle groups on 2 or more days a week (https://go.coe.int/IySxw). Total activity includes physical activity accrued at work, leisure, home or during transportation (Bull et al., 2020), but in prisoners these components have a much less impact on overall physical activity than in the rest of society. This means that in order to maintain health, prisoners have greater needs to participate in organized activities (training) resulting in a burden on the body in terms of aerobic physical activity and bone-strengthening activity. Prisoners are exposed to serious health effects of insufficient physical activity, well described in the literature (Woods, Breslin & Hassan, 2017). There are few scientific reports on motor organ dysfunction in prisoners (Minayo & Ribeiro, 2016; Nolan & Stewart, 2017). However, considering not only the significant shortcomings of physical activity among prisoners, but also the multi-faceted disadvantageous health conditions in prison, there may be concerns that imbalances among inactive prisoners occur more often than among inactive non-incarcerated persons. Establishing this could become the basis for incorporating measures to prevent pathological changes in the balance organ into wider strategies to reduce the negative health effects of imprisonment.

The aim of the study was to analyze postural stability of physically inactive prisoners in comparison to clinical control group consisting of physically non-active free (not imprisoned) citizens.

In available literature, similar studies have not been not conducted so far.

## Material and Methods

The research was conducted following the declaration of Helsinki. After obtaining consent to conduct the study from the Józef Piłsudski University of Physical Education Senate Commission of Ethics of Scientific Research no SKE-01-35/2015, the study group consisted of 24 male prisoners (oral agreement to participate in the study) aged 34.6 ± 7.02 years, imprisoned in a closed prison.

We compared results to those obtained from a control group aged 36.9 ± 7.5 years. The control group consisted of 30 healthy, non-active physically (not engaged in any physical activity except sedentary occupation for min. 5 years), age-matched men (oral agreement to participate in the study). For the highest reliability of statistical analyses, the groups were balanced for age, height, and body mass.

Table 1 presents the anthropometric data of the study and control group. There were no significant differences between the groups. The physical activity level of participants was assessed by questionnaire. The questionnaire included questions about taking up physical activity alone (in a cell or on a walking square) and as part of activities organized by the prison administration. The results of the questionnaire on activity in formal groups were compared with the information contained in the Central Database of Persons deprived of their liberty (employment, consent to exercise, membership in activity groups, personal identification notes). Initially, the use of a validated questionnaire to assess physical activity was considered, expecting prisoners to answer questions relating to activities not available to them may be a source of additional suffering in the form of considerable discomfort, deeper experience of deprivation of needs and thus lower mood or frustration. Given this argument, a decision was made to create a short questionnaire enabling the identification of physically inactive prisoners, adapted to the needs of the research.

**Table 1:** The anthropometric data of the study and control group.

Group		Age	Body height	Body mass
Prisoners (*N* = 24)	Mean	34.64	181.44	82.68
	Standard deviation	7.02	5.32	10.81
Control (*N* = 30)	Mean	36.87	179.50	74.56
	Standard deviation	7.46	8.30	32.96
Total (*N* = 54)	Mean	35.85	180.38	78.70
	Standard deviation	7.28	7.11	24.41

**Note:**

None of the differences has been significant on the 0.05 level.

The organizational possibilities have limited the sample size. The study was associated with the need not only to obtain the consent of the prison authorities but also to ensure full voluntary participation. The current sample size, with assumed test power 0.8, allows detection effects stronger than 0.7.

The criteria for prisoner’s inclusion were as follows: persons describing themselves in the questionnaire as physically inactive, who have never participated in regular sports activities in prison (did not obtain consent or did not seek consent), who, moreover, for the last 0.5 years, did not participate in a therapeutic or rehabilitation program containing some elements of physical activity, but could have participated in such programs earlier (*e.g*. once a week during the 8-week addiction therapy), age 20–60, verbal consent to participate in the study.

The subjects were imprisoned for a mean of 105.4 ± 58.5 months, 26 prisoners (89.7%) did not undertake employment, and 3 prisoners had physical jobs.

We excluded men’s with the following characteristics: prisoners who did not express informed consent to participate in the study. Exclusion criteria for prisoners and control group were as follows: cranial and cerebral injuries, visual system disorders, ear and sinus infections, low back pain, other injuries to the lower extremities, chronic diseases (*i.e*. cancer, Parkinson’s disease, epilepsy, diabetes, neuromuscular disorders, unsupervised coronary thrombosis), and taking psychoactive substances, *e.g*. participation in a substitution methadone program (Truszczyńska-Baszak, Drzał-Grabiec & Tarnowski, 2021).

Inclusion criteria for the control group were male, aged 20–60 sedentary and non-regular leisure-domain physical activity from min. 5 years.

**Figure 1:**
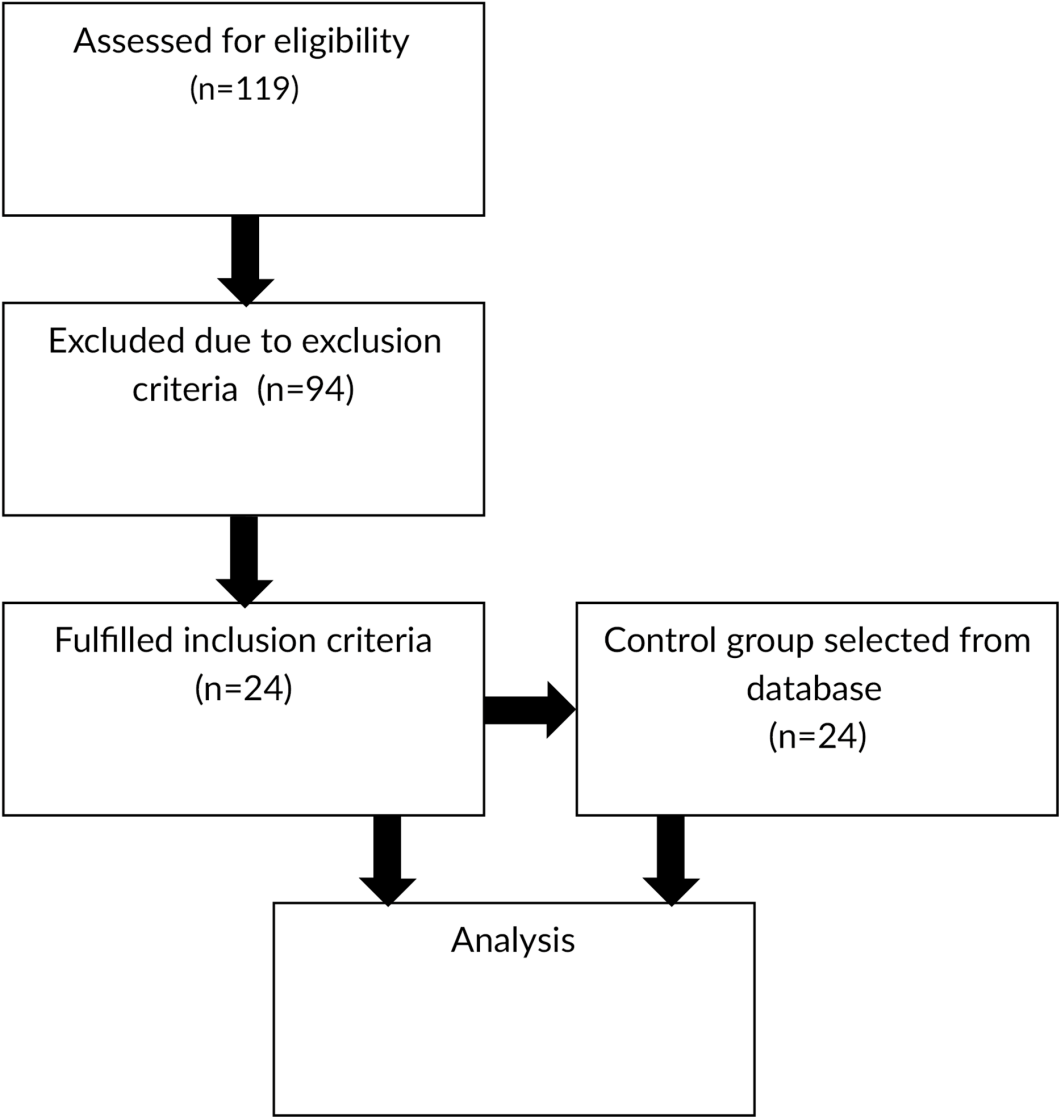
Diagram flow of participants.

Diagram flow of participants is presented on Fig. 1.

**Figure 2:**
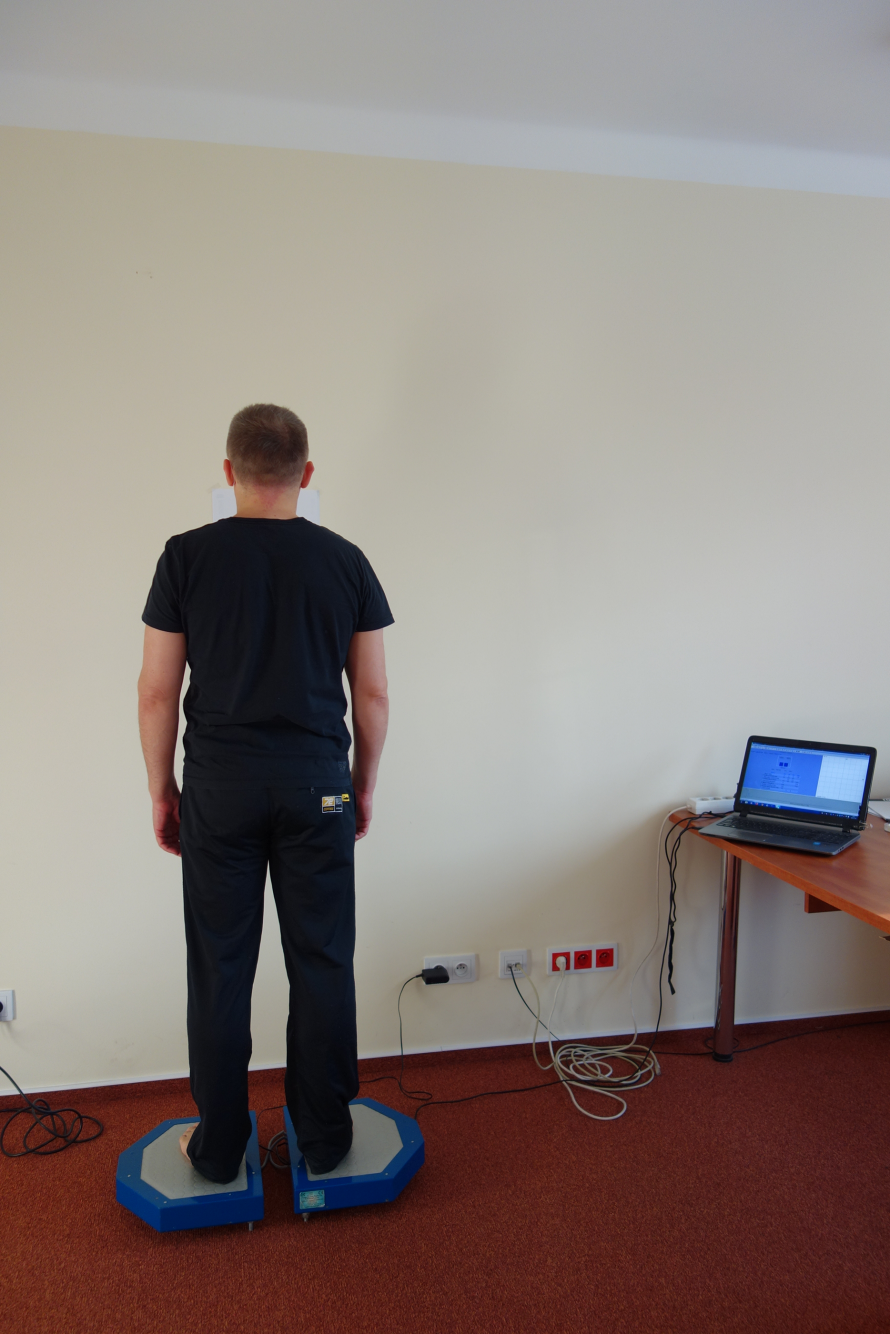
Presentation of measurement equipment- bi-modular stabilometric platform CQStab2P.

The static balance test was conducted on bi-modular stabilometric platform CQStab2P, registering the movement of centre of foot pressure (COP) (Fig. 2). It is a reliable measurement tools, its measurement error is 0.86% (www.cq.com.pl/n_platforma.htm).

Before joining the study protocol, the participants had been informed on the aim of the conducted measurements, their detailed course, and the possibility to resign at any chosen moment. Data were collected as previously described in Truszczyńska-Baszak, Drzał-Grabiec & Tarnowski (2021).

We analysed the parameters with both eyes open or closed:

**SP **– the statokinesiogram path (the projection of COP) (mm)
**SPAP **– the statokinesiogram path along the *y*-axis (mm)
**SPML **– the statokinesiogram path along the *x*-axis OX (mm)
**MA **– the mean amplitude of COP in two dimensional system of coordinates (mm)
**MAAP **– the mean amplitude of COP in the anterior-posterior direction (mm)
**MAML **– the mean amplitude of COP in the medio-lateral direction (mm)
**MaxAP **– the maximum distance between two furthest points in the anterior-posterior direction (mm)
**MaxML **– the maximum distance between two furthest points in the medio-lateral direction
**MV **– mean COP velocity
**MVAP **– mean COP velocity on the *y*-axis (mm/s)
**MVML **– mean COP velocity on the *x*-axis (mm/s) (Truszczyńska-Baszak et al., 2020; Drzał-Grabiec & Truszczyńska-Baszak, 2020)

The test was carried out in the morning hours in a specially prepared room. We made sure that the measurements were not disrupted by any noise from the outside. The subject was asked to rest seated for 5 min before testing. During that time, the equipment was being disinfected and calibrated. Then, the subject was asked to remove footwear and socks and to stand on both feet, hip width apart, arms along the body and eyes looking forward (Fig. 2). There were two 30 s long trials, the first with eyes open and the second with eyes closed. We had to use a procedure that based on single measurement due to prison’s organization limitations (lack of time, security, prisons attitude) (Truszczyńska-Baszak, Drzał-Grabiec & Tarnowski, 2021). There are several up to date studies that used this protocol in cases were triple measurements could not be used due to subjects health limitations (Truszczyńska-Baszak et al., 2020; Truszczyńska-Baszak, Drzał-Grabiec & Tarnowski, 2021; Drzał-Grabiec & Truszczyńska-Baszak, 2020).

### Statistical analysis

Statistical analysis has been performed using SPSS v. 27.

We have validated the integrity of data by checking the range of registered parameters. In all variables (including recording with eyes closed and open) range was smaller than six standard deviations, and maximum/minimum values were comparable to obtained in other experiments. Those argument ensured us about absence of robust artifacts. There were, however, some skewness coefficients larger than 1(−1) -in most cases such situation was observed in both groups. Due to small sample size we avoided to eliminate or transform data, we decided to use nonparametric analysis instead.

Group differences in controlled anthropometric variables have been tested using the Mann-Whitney rank test, and no significance has been found (Tab. 1).

The study aimed to explore differences between imprisoned people and a general population.

The Kolmogorov–Smirnov test has been implemented to assess the normality of posturographic variables and determine the methods of statistical analyses. Eleven of 44 distributions were recognized as non-normal, so the nonparametric methods were used to avoid ambiguous interpretation. However, SP distributions with eyes open and closed were normal-so cluster analysis for these variables was possible.

The averages of posture indicators have been compared. Table 2. Presents statistics of parameters registered with eyes open, Table 3. with eyes closed, and Table 4 – Mann–Whitney test results.

**Table 2:** Differences between prisoners and general population in stability parameters registered with eyes open.

Group		SP-EO (mm)	SPAP-EO (mm)	SPML-EO (mm)	MA-EO (mm)	MAAP-EO (mm)	MAML-EO (mm)	MaxAP-EO (mm)	MaxML-EO (mm)	MV-EO (mm/s)	MVAP-EO (mm/s)	MVML-EO (mm/s)
Prisoners	Mean	157.68	112.24	86.56	1.96	1.84	0.46	6.31	1.86	5.26	3.75	2.89
	Std. Dev.	26.18	27.59	11.68	1.01	0.97	0.25	3.22	0.82	0.88	0.92	0.39
	Median	153.00	102.00	86.00	1.70	1.60	0.40	4.70	1.90	5.10	3.40	2.90
Control	Mean	128.57	97.57	63.43	1.79	1.53	0.65	5.22	2.19	4.29	3.26	2.13
	Std. Dev.	25.71	23.94	12.15	1.00	0.94	0.50	2.22	1.25	0.86	0.79	0.40
	Median	126.50	91.50	60.00	1.55	1.30	0.50	5.05	2.00	4.25	3.05	2.00
	U	154.50	254.50	70.00	336.50	295.00	290.50	322.00	334.50	157.50	254.50	72.50
	Z	−3.73	−2.04	−5.16	−0.652	−1.35	−1.44	−0.90	−0.69	−3.68	−2.04	−5.12
	Two-tail p	<.001	0.042	<0.001	0.515	0.175	0.149	0.370	0.493	<0.001	0.041	<0.001
	Effect size	0.25	0.08	0.48	0.01	0.03	0.04	0.01	0.01	0.25	0.08	0.48

**Table 3:** Differences between prisoners and general population in stability parameters with eyes closed.

Group		SP-EC (mm)	SPAP-EC (mm)	SPML-EC (mm)	MA-EC (mm)	MAAP-EC (mm)	MAML-EC (mm)	MaxAP-EC (mm)	MaxML-EC (mm)	MV-EC (mm/s)	MVAP-EC (mm/s)	MVML-EC (mm/s)
Prisoners	Mean	180.37	141.58	82.88	1.80	1.70	.39	5.90	1.58	6.00	4.73	2.77
	Std. Dev.	42.89	44.43	12.45	0.822	0.817	0.17	2.15	0.714	1.44	1.48	0.42
	Median	178.00	138.00	83.00	1.70	1.65	0.40	5.95	1.45	5.90	4.60	2.80
Control	Mean	196.43	167.37	73.00	2.12	1.94	0.57	7.75	2.60	6.55	5.57	2.43
	Std. Dev.	62.12	61.93	14.46	0.69	0.68	0.32	3.82	2.45	2.07	2.07	0.47
	Median	193.00	161.00	72.00	2.05	1.80	0.50	7.10	2.05	6.45	5.40	2.40
	U	324.50	275.50	213.00	260.50	279.00	194.50	253.00	205.00	320.50	277.50	205.00
	Z	−0.62	−1.47	−2.56	−1.74	−1.41	−2.94	−1.86	−2.70	−0.69	−1.44	−2.71
	Two-tail p	0.536	0.141	0.010	0.083	0.158	0.003	0.062	0.007	0.491	0.151	0.007
	Effect size	0.01	0.04	0.12	0.05	0.04	0.16	0.06	0.13	0.01	0.04	0.13

**Table 4:** SP statistics for SP parameter registered with eyes open and close in three empirical taxonomy groups.

Empirical taxonomy		SP-EO (mm)	SP-EC (mm)
A	Mean	123.33	150.85
	Std. Dev.	18.54	29.07
	N	27	27
B	Mean	175.38	209.69
	Std. Dev.	17.47	27.59
	N	16	16
C	Mean	134.82	254.00
	Std. Dev.	21.04	56.26
	N	11	11
Total	Mean	141.09	189.30
	Std. Dev.	29.36	54.54
	N	54	54

Six differences proved to be significant with eyes open (SP, SP-AP, SP-ML,MV, MV-AP and MV-ML) when only four with eyes closed (SP-ML, MA-ML, MAX-ML, MV-ML).

**Figure 3:**
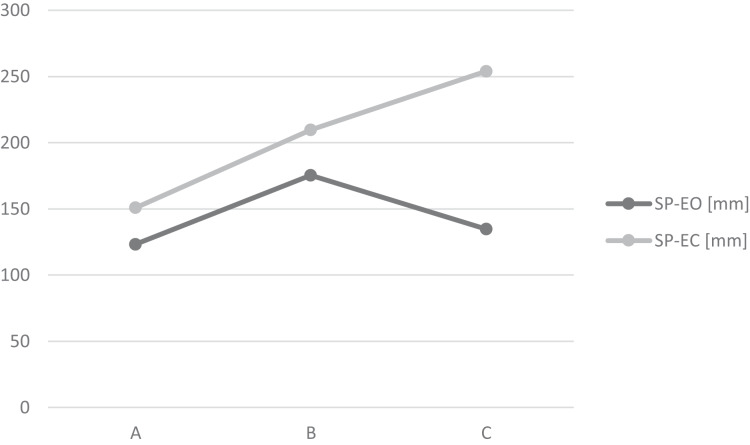
Mean values of SP for three empirical taxonomy groups.

Hierarchical cluster analysis has been implemented to explore possible qualitative relations based on two general and representative variables (SP with eyes open and with eyes closed). Data have been transformed using z-standardisation, the Cosinus distance metric and the BAVERAGE linking method have been implemented. Finally, three groups have been identified as homogenous and numerous enough. Group characteristics are presented at Table 4 and Fig. 3.

The A group (A largest one; *N* = 27) is characterized by the best stability parameters, both with eyes open and closed. The B group (*N* = 16) is presenting diminished stability, also in both situations. The third group, C, (*N* = 11) demonstrate pretty good stability parameters with eyes open, but the worst with eyes closed (Table 5).

**Table 5:** The relationship between empirical taxonomy and imprisoning.

			Group		Total
			Prisoners	Control	
Empirical taxonomy	A	Frequency	11	16	27
		% z Empirical taxonomy	40.7%	59.3%	100.0%
	B	Frequency	11	5	16
		% z Empirical taxonomy	68.8%	31.3%	100.0%
	C	Frequency	2	9	11
		% z Empirical taxonomy	18.2%	81.8%	100.0%
Total		Frequency	24	30	54
		% z Empirical taxonomy	44.4%	55.6%	100.0%

The relationship between empirical taxonomy and imprisoning has been checked by using chi-square test.

**Figure 4:**
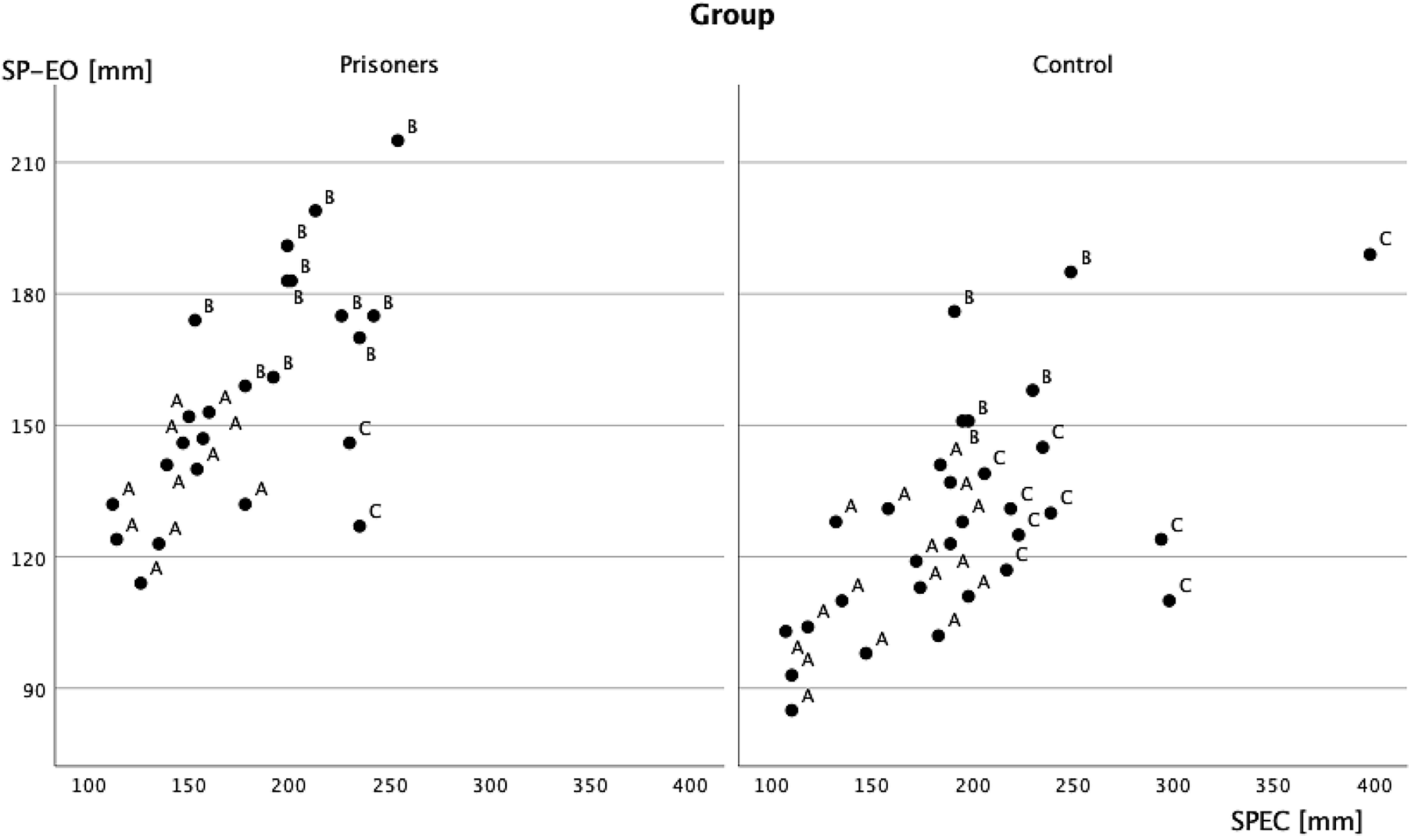
Scatterplot of SP with eyes open and closed in two groups, concerning empirical taxonomy.

The correlation between imprisoning and empirical taxonomy proved to be statistically significant (Chi-square = 7.05, df = 2 *p* < 0.05) The column proportions have been compared using *z*-test with Bonferroni correction for multiple comparisons. No difference has been found in A group, but B group were more frequent in the prisoners’ population, and the C group was in the controls (Fig. 4).

## Discussion

When the mechanisms for correcting the body’s balance are not constantly supported by exercise, they are likely getting worse. The models of deterioration, however, depends on the environment. All significant differences observed with eyes open (SP, SP-AP, SP-ML,MV, MV-AP and MV-ML) favorize the control group. An interesting relationship can be observed in parameters registered with eyes closed: SP-ML, MV-ML are better in the general population sample, but MA-ML and MAX-ML are better in the prisoners’ group. All dependencies are observed in the mediolateral plane; when in the anterior-posterior axis, no significant differences are observed. So, the corrective stability movements in the prisoner’s group are faster but effective. The effect should be treated as post-hoc observation, so the model is still hypothetical and incomplete.

The empirical taxonomy approach allows for one more hypothetical explanation. According to it, there are no differences between prisoners and members of general, physically inactive population whose compensatory mechanisms are efficient. The lack of efficiency in the prisoners’ group manifests itself in the reduction of all parameters. In contrast, in the general population sample, the lack of fitness mainly affects the mechanisms based on proprioception and the functioning of the sense of balance, which are essential for stabilizing the body posture with eyes closed.

The specific of relationship between body balance and imprisoning should be also commented in context of theoretical models. Several sensory systems are involved in maintaining body balance. Consequently, the range of diseases which may result in dizziness and balance disorders is broad (Gandolfi et al., 2018). The disorders may result from a neurodegenerative disease, or from some infections, *e.g*. of the brain or of the inner ear. As taking some medicines may also result in balance disorders (Hohler & de Leon, 2018), it is also possible that treating illnesses unrelated to the balance system may require using drugs that cause balance disorders.

One of the causes of balance disorders may be the decreased effectiveness of the motor or locomotor system (Van Nechel, 2017), including that resulting from trunk or leg muscle strength deficits (Hohler & de Leon, 2018). Such state may be caused by insufficient physical activity. Insufficient physical activity may result in changes to even the simplest motor activities, such as sitting straight or walking (Shibkova & Alyonin, 2018).

All above mentioned possible causes of balance disorders are overrepresented in prisoners. This may partly explain the higher level of balance disorders in studied prisoners in comparison to the control group. Prison environment is widely believed to be unfavourable for health and prisoners manifest numerous behaviours that may negatively affect their health (Enggist et al., 2014). In effect, non-contagious diseases (NCDs) are disproportionally more common among prisoners (as well as among other disadvantaged social groups) (Herbert et al., 2012). Mental health disorders, such as psychosis, depression, personality disorders or post-traumatic disorder (Fazel & Danesh, 2002) and dementia (Forsyth et al., 2020) are much more common in prisoners that in the general public. Many sources indicate that imprisonment results in insufficient physical activity (Battaglia, di Cagno & Fiorilli, 2013; Fazel & Baillargeon, 2011; Stoever et al., 2007). Insufficient physical activity may be related to chronic spinal disorders, back pain as well as other musculoskeletal disorders reported by prisoners (Report to the Polish Government on the visit to Poland carried out by the European Committee for the Prevention of Torture and Inhuman or Degrading Treatment or Punishment (CPT), 2017; Minayo & Ribeiro, 2016; Moxey-Adderley et al., 2019; Stewart et al., 2015).

Another explanation of differences between the prisoners and the control groups is the prisoners’ early ageing, already described in the literature (Bedard, Metzger & Williams, 2016). Prisoners are one of those social groups in which some age-related diseases develop even 15 years earlier than in the general population. This is the reason why the definition of old age in relation to prisoners uses the threshold of 50 or 55 years of age (Aday, 2003; Williams & Abraldes, 2007).

Dizziness is the most common cause of falls. Health-related consequences of falls are usually more severe in older adults because of, *inter alia*, the possible changes to the bone structure and age-related slower reaction time (Edbom-Kolarz & Marcinkowski, 2011; Sterling, O’Connor & Bonadies, 2021).

The analysis of our results found balance disorders in the sagittal plane, which predisposes the prisoners to fall to the side. The range of sways seems early for their age. The conclusion that follows is that the age-related increased risk of falls is prevalent in prisoners relatively younger than in non-imprisoned subjects. In prisons therefore prevention of falls of older adults should be implemented in relatively younger population. The necessity of implementing prevention of falls in prisons is supported by the data on the ageing of the worldwide prisoner population (Shaw, 2020), a trend that is also present in Poland. The complex falls prevention in prisons should widely extend the actions of the healthcare, and it should also involve relevant architectonic solutions and equipment of the rooms and places where prisoners spend their time (Bedard, Metzger & Williams, 2016; UNOPS, 2016).

Appropriate management of the health of the older prisoner population requires adequate diagnostics. Screening tests for NCDs in prisoners are necessary. The WHO reports that its member states will need to implement screening tools for full management of NCDs and related factors (WHO Regional Office for Europe, 2019). The older prisoner population requires additional screening tests, however (Atabay, 2009). Monitoring of imbalances in older prisoners is also needed, as typical geriatric conditions (including dizziness and falls) are often underdiagnosed and their detection is necessary to assess the etiology of functional limitations in frail elders (Liu & Walter, 2014).

### Limitations of the study

The study was conducted in a single center with a small sample size, and only one postural stability measurement was performed so results should be generalized with caution.

In Poland there are no significant differences in terms of the standard of living space, duration of the walk and other issues affecting the involvement of motor skills in everyday activities. Therefore, it can be concluded that the group of respondents is representative of closed type prisons in Poland.

Second, this study only showed a cross-sectional association between balance disorders status and inactivity. The causal relationship between the results is unknown.

The presented study does not provide enough essential data to implement balance disorder prevention measures in prisons, as it does not present the reasons for these disorders. The possible reasons for balance disorders were only presented in the analysis of the literature. As the balance mechanism is complex it is necessary to conduct a study which would find the most common causes for balance disorders in prisoners.

It shall be also noticed that statistical differences are significant, but the effect size are not reaching assumed values. Thus, the results are vulnerable for second type error, and before confirmation on larger sample should be treated as hypothesis. However, the data merits attention, and proposed model should be considered.

The directions of further studies. Counteracting hypokinesis should be based on the rule that all the imprisoned persons are able to do physical exercise on their own in their free time. The ability itself may not be sufficient, due to the low healthcare awareness of some prisoners. This is why prisons should take healthcare education actions that would involve motivating for exercise and instructions that would enable prisoners to perform exercise correctly. It should also be possible to refer certain prisoners to physical therapy in case of disorders caused by hypokinesia.

## Conclusions

Diagnostics aimed at early diagnoses of ageing symptoms including postural stability assessment should be performed in prisons. It would allow for better prisoner management in terms of assessment of ability to work, free time activity offer, falls prevention *etc*. An element of such diagnostics should be the monitoring of body balance disorders.

## Supplemental Information

10.7717/peerj.12489/supp-1 Supplemental Information 1 Raw data of all examined prisoners.

## Additional Information and Declarations

### Data Availability

The following information was supplied regarding data availability: The raw data is available in the Supplemental File.
